# Construction of a Competitive Endogenous RNA Network and Identification of Potential Regulatory Axis in Gastric Cancer

**DOI:** 10.3389/fonc.2019.00912

**Published:** 2019-10-04

**Authors:** Hongda Pan, Chunmiao Guo, Jingxin Pan, Dongwei Guo, Shibo Song, Ye Zhou, Dazhi Xu

**Affiliations:** ^1^Department of Gastric Surgery, Fudan University Shanghai Cancer Center, Shanghai, China; ^2^The Second Affiliated Hospital of Fujian Medical University, Quanzhou, China; ^3^Department of Oncology, Shanghai Medical College, Fudan University, Shanghai, China; ^4^State Key Laboratory of Ophthalmology, Zhongshan Ophthalmic Center, Sun Yat-sen University, Guangzhou, China; ^5^Department of Gastrointestinal Surgery, Beijing Hospital, Beijing, China

**Keywords:** gastric cancer, bioinformatics analysis, competitive endogenous RNA, prognostic biomarker, experimental validation

## Abstract

**Background:** Increasing studies has found that long non-coding RNAs (lncRNAs) play critical roles in carcinogenesis, but the underlying mechanisms remain unclear. The aim of this study is to construct a competitive endogenous RNA (ceRNA) network and to identify potential regulatory axis in gastric cancer (GC).

**Methods:** Differentially expressed (DE) mRNAs, miRNAs, and lncRNAs were obtained by analyzing the RNA expression profiles of stomach adenocarcinoma (STAD) retrieved from The Cancer Genome Atlas (TCGA) database. The lncRNA-miRNA-mRNA regulatory networks of GC were constructed by comprehensive bioinformatics methods including functional annotation, RNA-RNA interactomes prediction, correlation analysis, and survival analysis. The interactions and correlations among ceRNAs were validated by experiments on cancer tissues and cell lines.

**Results:** A total of 41 lncRNAs, 9 miRNAs, and 10 mRNAs were identified and selected to establish the ceRNA regulatory network of GC. Several ceRNA regulatory axes, which consist of 18 lncRNAs, 4 miRNAs, and 6 mRNAs, were obtained from the network. A potential ADAMTS9-AS2/miR-372/CADM2 axis which perfectly conformed to the ceRNA theory was further analyzed. qRT-PCR showed that ADAMTS9-AS2 knockdown remarkably increased miR-372 expression but reduced CADM2 expression, whereas ADAMTS9-AS2 overexpression had the opposite effects. Dual luciferase reporter assay indicated that miR-372 could bound to the ADAMTS9-AS2 and the 3′UTR of CADM2.

**Conclusion:** The constructed novel ceRNA network and the potential regulatory axes might provide a novel approach of the exploring the potential mechanisms of development in GC. The ADAMTS9-AS2/miR-372/CADM2 could act as a promising target for GC treatment.

## Introduction

Long non-coding RNAs defined as non–protein-coding RNAs longer than 200 nucleotides (1). lncRNAs are abundant in mammalian cells, associated with regulation of gene expression in carcinogenesis. Recently, a great many of reports have shown that lncRNAs serve as therapeutic targets and prognostic indicators (2–4).The ceRNA hypothesis has been proposed that lncRNAs may regulate other RNA transcripts by competing for shared microRNAs (5), which play a critical role in the progression of cancers (6, 7).

Recently, high throughput sequencing technologies has provided oncologists with a powerful tool to identify potential biomarkers for diagnosis and treatment of cancer. The comprehensive bioinformatics methods have been applied for oncology studies, which greatly facilitate the exploration of a great amount of important biological data. According to the hypothesis of ceRNA, lncRNA-miRNA-mRNA networks have been constructed in several types of cancer (8–15). Nevertheless, these studies failed to verify the reliability of their findings, and the algorithms were ambiguous and unreasonable.

In the present study, we analyzed the aberrantly expressed lncRNA, miRNA, and mRNA in GC samples from TCGA database. In order to provide a comprehensive view of regulatory mechanism of GC, the aberrant lncRNA-miRNA-mRNA network was constructed by comprehensive bioinformatics approaches. To validate the results obtained from bioinformatics analysis, the expression of key RNAs in GC cell lines and clinical samples were detected by quantitative real-time PCR (qRT-PCR) and dual luciferase reporter assay.

## Materials and Methods

### TCGA Data Retrieval

Expression profiling data of 443 GC patients were retrieved from TCGA-STAD database. The GDC Data Transfer Tool was used to download the level 3 mRNASeq gene expression profiles (including 375 GC tissues and 32 adjacent normal gastric tissues), miRNAseq data (including 446 GC tissues and 45 adjacent normal gastric tissues), and corresponding clinical data was also obtained from TCGA-STAD dataset (up to June 13, 2018). The Ensembl database (16) was used to annotate the lncRNAs and mRNAs, RNAs which were not included in the annotation list were then discarded. The RNA expression profiling data is already normalized in TCGA database, therefore secondary normalization was unnecessary. This part of our study was performed according to the publication guidelines of TCGA, and no approval was required from the local Ethics Committee.

### Identification of DERNAs

To identify the DE lncRNAs, miRNAs, and mRNAs in GC in comparison with normal tissue, the expression profiles were analyzed using the edgeR package (17), which is based on R language. A false discovery rate (FDR) was applied for identifying differentially expressed RNAs. FDR <0.01 and fold changes (log_2_ absolute) ≥2 were considered statistically significant. Volcano plots and Heatmaps were generated by the “ggplot2” and “pheatmap” packages in R software to demonstrate differentially expressed RNAs.

### Functional Annotation

To illustrate functional annotations implicated with the DEmRNAs, gene ontology (GO) annotation and Kyoto Encyclopedia of Genes and Genomes (KEGG) pathway analysis were carried out by “clusterProfiler” package in R software (18). The top 5 significant biological processes or pathways were visualized by Circos plots created by “ggplot2” package.

### Construction of a ceRNA Network

**Figure 1:**
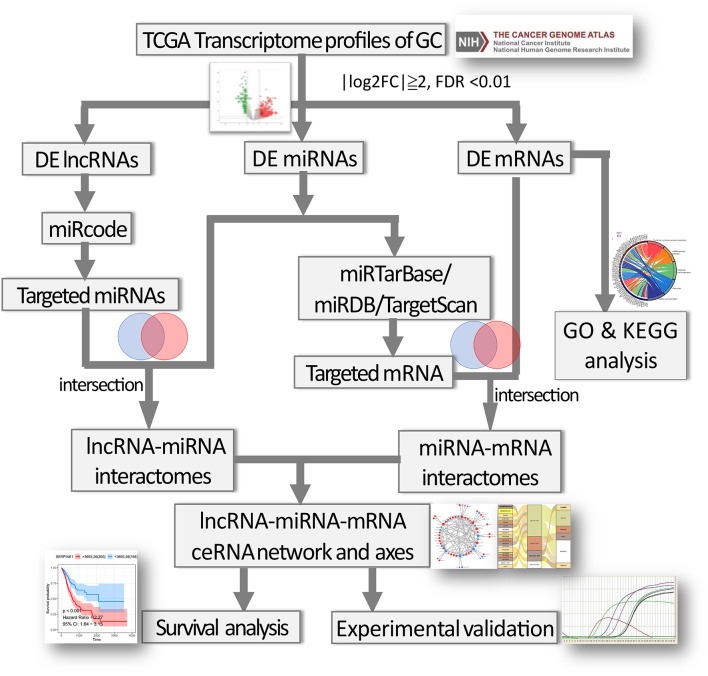
Flow chart of the construction of ceRNA network and regulatory axes in gastric cancer.

The flow chart for the ceRNA network construction was showed in Figure 1. First, the file of putative interactomes of lncRNA-miRNA were downloaded from miRcode (19). By retrieving the names of differentially expressed lncRNAs (DElncRNAs) in the miRcode database, we can predict the lncRNA-miRNA interactomes. The target miRNAs were then intersected with DEmiRNAs. Next, miRTarBase (20), miRDB (21), and TargetScan (22) were utilized to predict miRNA-mRNA interactomes and retrieve experimentally validated data. The target mRNAs were further intersected with DEmRNAs. The ceRNA network was subsequently generated by combining the lncRNA-miRNA interactomes and miRNA-mRNA interactomes. The Cytoscape software (v3.6.1) (23) was used to visualize the ceRNA networks, and the hub gene and sub-network was identified by “cytoHubba” plugin. According to ceRNA hypothesis, miRNA expression was negatively correlated with lncRNA or mRNA. Thus, for identification of potential ceRNA regulatory axes, the positively correlated lncRNA-miRNA pairs and miRNA-mRNA pairs in the ceRNA network were discarded. R package “ggalluvial” was used to demonstrate the ceRNA axes.

### Clinical Samples

Fifty-four GC and paired normal gastric tissues were collected from biobank of Beijing Hospital. Written informed consent was obtained from all patients. This study was performed with the approval of the Ethics Committee of the Beijing Hospital.

### Cell Culture and Transfection

Human gastric epithelial cell line (GES-1) and GC cell lines (MKN-45 and HGC-27) were purchased from Health Science Research Resources Bank. Cell were cultured in DMEM (Gibco, Rockford, MD, USA) supplemented with 10% FBS at 37°C in an atmosphere of 5% CO_2_. ADAMTS9-AS2 siRNA, pcDNA3.1 vector and miRNA mimics (GenePharma, Shanghai, China) were transfected by Lipofectamine 3000 (Invitrogen, USA).

### Quantitative Real-Time PCR (qRT-PCR)

TRIzol reagent (Thermo Fisher Scientific, Carlsbad, CA, USA) was used to extracted the total RNA from GC cells, tissues, and normal tissues according to the manufacturer's protocol. Reverse transcription was performed using a Prime Script RT reagent kit (Takara Biotechnology, China). Applied Biosystems 7900 Real-time PCR Systems (Thermo Fisher Scientific) was used to perform the qRT-PCR assay. GAPDH was used to normalize lncRNA and mRNA expression and small RNA RNU6 (U6) was used for miRNA. Primers used for amplification of targets were shown in Table S1.

### Western Blotting Analysis

Protein was extracted from GC cells with RIPA lysis buffer (Thermo Scientific, USA). Identical quantities of proteins were electrophoresed by SDS-PAGE (Life Technology, USA), and transferred onto PVDF membranes (Millipore, USA). After that, total proteins were incubated with primary antibodies at 4°C overnight. The target protein was detected with a polyclonal antibody (Abcam, USA). Then, the membrane was washed with PBS *three* times and incubated with secondary antibody. GAPDH (CST, USA) was used as standard loading control.

### Fluorescence *in situ* Hybridization (FISH)

Cell nuclei were counterstained with DAPI. To determine the co-localization of ADAMTS9-AS2 and miR-372 in GC cells, Cy5-labeled ADAMTS9-AS2 probes and Cy3-labeled miR-372 probes were used for a double FISH assay. A Fluorescent *in situ* Hybridization Kit (Gene-Pharma, China) was used following the manufacturer's protocols.

### Dual Luciferase Reporter Assay

The 3′-UTR sequences of CADM2 or ADAMTS9-AS2 that including wild-type or mutant miR-372 binding sites were synthesized. MKN-45 cells were co-transfected with ADAMTS9-AS2/CADM2 3′UTR reporter plasmids (wt or mut) luciferase plasmids and miR-372 mimics or miR-NC. After 48 h of transfection, luciferase activities were detected with a Dual-Luciferase Reporter Assay System (Promega, Madison, WI, USA). Firefly luciferase was normalized to Renilla luciferase for individual well.

### Survival and Statistical Analysis

After downloading the clinical data from TCGA, the “survival” package in R software was used for survival analysis. A “survminer” package in R software to determine the best cut-off of the expression value for survival analysis. Correlations between the expression of RNAs were analyzed by Pearson correlation analysis. The two-tailed Student's *t*-test was used to compare the differences of expression level between groups. The correlations between expression level and clinicopathologic parameters were determined using chi-square test. A *p* < 0.05 was considered statistically significant.

## Results

### Differentially Expressed RNAs (DERNAs) in GC

**Figure 2:**
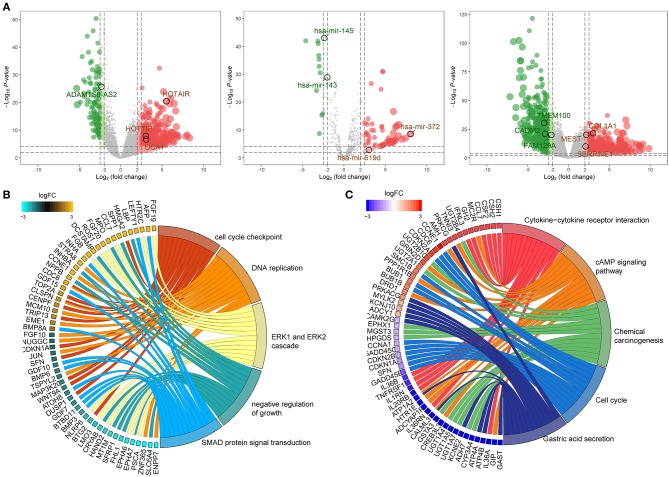
Identification of differentially expressed RNAs and functional annotation of DEmRNA. **(A)** Volcano map of differentially expressed lncRNA (left), miRNA (middle), and mRNA (right). Red and green spots represent significant up- and down-regulated RNAs, respectively. **(B)** The top 5 significantly enriched GO biological process and relevant genes. **(C)** The top 5 significantly enriched KEGG pathways and relevant genes.

One thousand and twenty-two DElncRNAs, 104 DEmiRNAs and 1,632 DEmRNAs were identified from TCGA-STAD database. Of these, 805 (78.8%) lncRNAs, 87 (81.3%) miRNAs, and 855 (52.4%) mRNAs were upregulated, and 217 (21.2%) lncRNAs, 17 (18.7%) miRNAs, and 777 (47.6%) mRNAs were downregulated in GC compared with normal tissue. The distributions of differentially expressed RNAs were demonstrated through volcano plots (Figure 2A).

### GO and KEGG Pathway Analysis of DEmRNAs

To investigate the potential functional implication of the 1,632 DEmRNAs, we performed GO and KEGG annotation of for DEmRNAs (Figures 2B,C). The DEmRNAs were primarily enriched in biological processes (BP) that associated with carcinogenesis, for instance, “cell cycle checkpoint,” “DNA replication,” “ERK1 and ERK2 cascade,” “negative regulation of growth,” and “SMAD protein signal transduction” (Figure 2B). In addition, KEGG mapping revealed that the remarkably enriched pathways are the “Cytokine-cytokine receptor interaction,” “cAMP signaling pathway,” “Chemical carcinogenesis,” “Cell cycle,” and “Gastric acid secretion,” which are related to the progression of GC (Figure 2C).

### Construction of a ceRNA Network in GC

**Figure 3:**
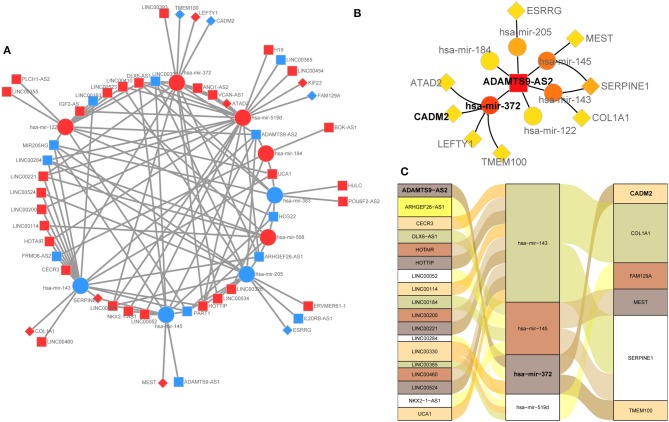
**(A)** Overview of the ceRNA network in gastric cancer. Up-regulation was represented by ed nodes, while down-regulation was represented by the blue nodes. round rectangle, ellipse, and diamonds represent lncRNAs, miRNAs, and mRNA, respectively. **(B)** lncRNA ADAMTS9-AS2 is a hub gene that directly interacted with 6 miRNAs, and indirectly interacted with 8 mRNAs. **(C)** lncRNA-miRNA-mRNA regulatory axes extracted from the ceRNA network.

The lncRNA-miRNA-mRNA network of GC was constructed by integrated analysis of the above results. The regulatory network was comprised 115 edges among 41 lncRNAs, 9 miRNAs, and 10 mRNAs (Figure 3A). We noted that the 6 DEmRNAs, namely SERPINE1 (24), COL1A1 (25), MEST (26), CADM2 (27), TMEM100 (28), and FAM129A (29), have been reported to be cancer-related genes. Moreover, the lncRNA ADAMTS9-AS2 may act as a hub gene for that it directly interacted with 6 miRNAs (hsa-mir-122, hsa-mir-143, hsa-mir-145, hsa-mir-184, hsa-mir-205, and hsa-mir-372) and then indirectly interacted with 8 miRNA-targeted mRNAs (SERPINE1, COL1A1, MEST, ESRRG, LEFTY1, TMEM100, ATAD2, and CADM2) in this ceRNA network (Figure 3B). We selected the negative interactomes from the ceRNA network to construct regulatory axes, which including 18 lncRNAs, 4 miRNAs, and 6 mRNAs (Figure 3C).

### Prognostic Significance of RNAs in the ceRNA Network

**Figure 4:**
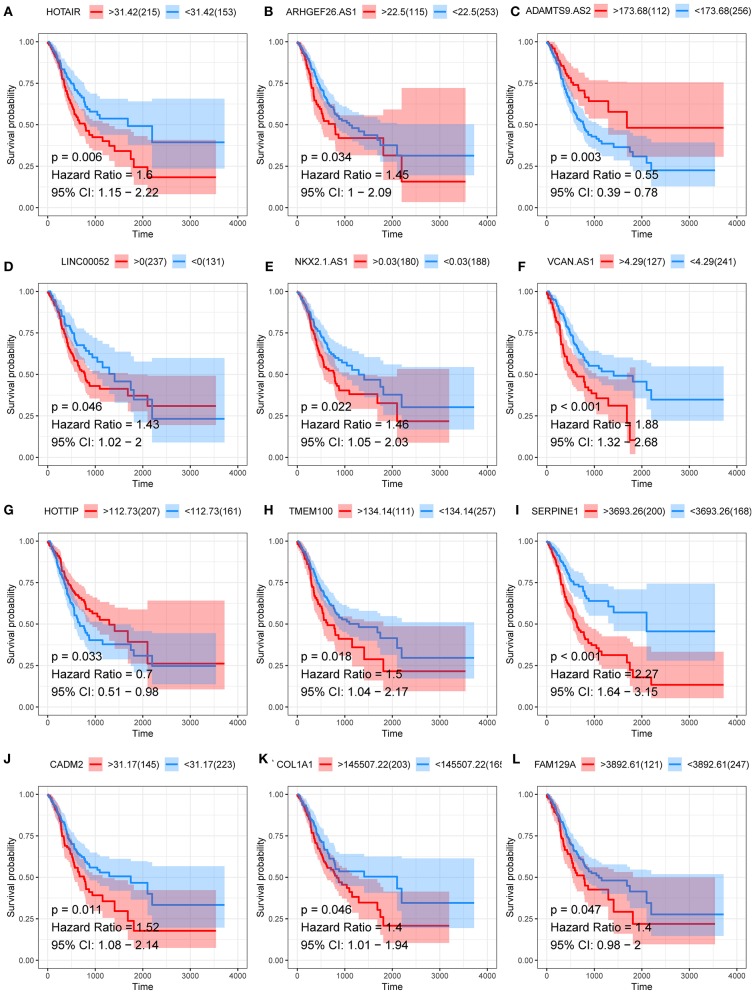
Kaplan–Meier curve of lncRNAs **(A–G)** and mRNA **(H–L)** that significantly correlated with overall survival.

The correlation between DERNAs and the survival outcomes of patients with GC was analyzed to discover the prognostic factors. The results show that 7 lncRNAs (ADAMTS9-AS2, ARHGEF26-AS1, HOTAIR, HOTTIP, LINC00052, NKX2-1-AS1, and VCAN-AS1), and 5 mRNAs (CADM2, COL1A1, FAM129A, SERPINE1, and TMEM100) were significantly correlated with overall survival (Figure 4).

### Correlation Between lncRNA and mRNA From the ceRNA Network

**Figure 5:**
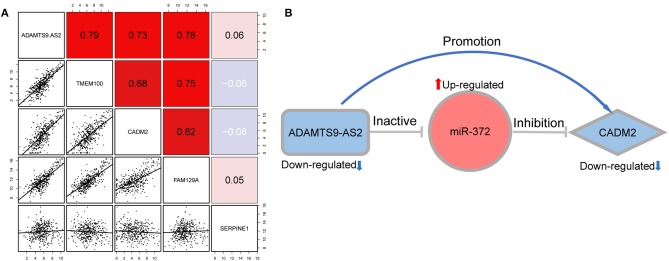
**(A)** Correlation between lncRNA ADAMTS9-AS2 and four mRNA (TMEM100, CADM2, FAM129A, and SERPINE1). **(B)** The ADAMTS9-AS2/ miR-372/ CADM2 regulatory axis which perfectly conformed to the ceRNA hypothesis.

On the basis of the hypothesis of ceRNA, lncRNA indirectly regulate mRNAs expression with a positive correlation. We analyzed the correlation between the lncRNA and mRNA in the constructed network. The results revealed that the expression of ADAMTS9-AS2 was strongly correlated with those of CADM2 (*r* = 0.73, *p* < 0.01), TMEM100 (*r* = 0.79, *p* < 0.01) and FAM129A (*r* = 0.78, *p* < 0.01) (Figure 5A).

### Identification of a Potential Regulatory Axis

ADAMTS9-AS2/miR-372/CADM2 was identified as a potential regulatory axis from the ceRNA network for the following reasons: First, ADAMTS9-AS2 and CADM2 were significantly downregulated, while miR-372 was significantly upregulated in GC compared with normal tissue. Second, our survival analysis showed that low expression levels of both ADAMTS9-AS2 and CADM2 were significantly associated with poor overall survival. Additionally, the interaction between ADAMTS9-AS2 and miR-372 was predicted by a highly reliable genomic database (miRcode); likewise, the interaction between miR-372 and CADM2 was confirmed by experimentally validated evidence (miRTarBase, miRDB, and TargetScan). Finally, correlation analysis revealed that the expression level of ADAMTS9-AS2 positively associated with that of CADM2. The above findings perfectly accorded with the “ceRNA hypothesis.” Therefore, we consider the ADAMTS9-AS2/miR-372/CADM2 to be a potential regulatory axis from the ceRNA network, which may exert a critical role in GC progression (Figure 5B).

### Experimental Validation

**Figure 6:**
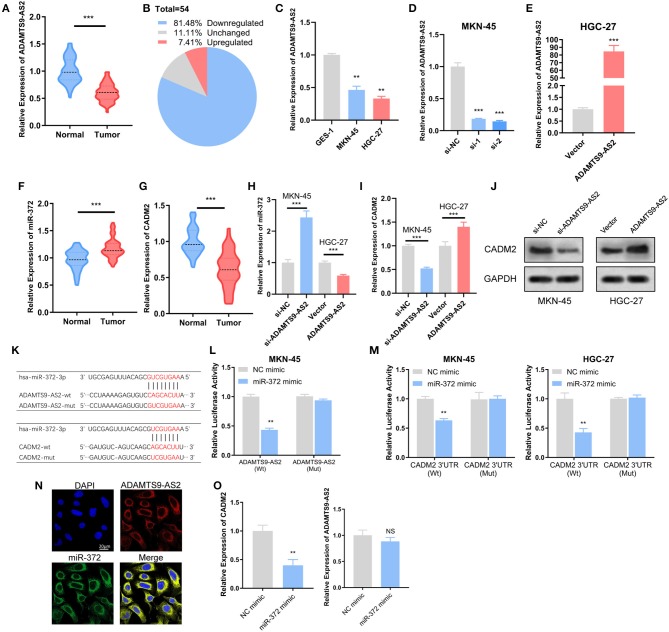
Experimental validation of ADAMTS9-AS2/miR-372/ CADM2 axis. **(A,B)** ADAMTS9-AS2 was downregulated in GC in comparison with normal tissues (*n* = 54). **(C)** ADAMTS9-AS2 was downregulated in MKN-45 and HGC-27 cells compared with gastric epithelial GES-1 cells. **(D,E)** ADAMTS9-AS2 was knockdown in MKN-45 cells and overexpressed in HGC-27 cells, the effects of knockdown and overexpression was measured by qRT-PCT. **(F,G)** miR-372 was upregulated and CADM2 was downregulated in GC compared with normal tissues (*n* = 54). **(H,I)** After knockdown or overexpression of ADAMTS9-AS2, the expression levels of miR-372 and CADM2 were measured in MKN-45 or HGC-27 cells. **(J)** The protein expression of CADM2 was determined after ADAMTS9-AS2 knockdown or overexpression by western blotting. **(K)** Schematic representation of the potential binding sites of miR-372 with ADAMTS9-AS2 and the 3′UTR of CADM2. **(L,M)** The luciferase activities after wild-type or mutant ADAMTS9-AS2 **(L)** or CADM2 3′UTR **(M)** luciferase reporter vector was co-transfected with miR-372 mimics into MKN-45 and HGC-27 cells. **(N)** FISH assay showed the co-localization of ADAMTS9-AS2 and miR-372. **(O)** The expression levels of ADAMTS9-AS2 and CADM2 after transfection of miR-372 mimic were measured by qRT-PCR. All experiments were performed in triplicate. Scale bar = 20 μm. ^*^p < 0.05, ^**^p < 0.01, ^***^p < 0.001, NS, non-significant.

The expression levels of ADAMTS9-AS2 were investigated in GC cell lines as well as in 54 paired GC and adjacent normal tissue samples. The expression of ADAMTS9-AS2 was down-regulated in 81.5% (44/54) of GC compared with those in normal tissues (Figures 6A,B). Additionally, low ADAMTS9-AS2 expression was associated with poor histologic differentiation (*p* = 0.004) and advanced TNM stage (*p* = 0.014) (Table 1). Next, we discovered that ADAMTS9-AS2 was underexpressed in two GC cell lines (MKN-45 and HGC-27), as compared to the normal gastric cell line (GES-1) (Figure 6C). The efficacy of knockdown and overexpression of ADAMTS9-AS2 in MKN-45 and HGC-27 were detected by qRT-PCR (Figures 6D,E).

**Table 1:** Correlation between ADAMTS-AS2 expression levels and the clinicopathological parameters of 54 GC patients.

**Clinicopathological parameters**	**Number of cases**	**ADAMTS9-AS2**		***P*-value**
		**High**	**Low**	
Sex				0.776
Male	35	17	18	
Female	19	10	9	
Age (years)				0.584
≥65	30	16	14	
<65	24	11	13	
Histologic differentiation				0.004
Well or moderate	36	23	13	
Poor	18	4	14	
TNM stage				0.014
I–II	29	19	10	
III–IV	25	8	17	
Serum CEA level				0.785
>5 ng/ml	25	12	13	
≤5 ng/ml	29	15	14	
Lymphovascular invasion				0.068
Negative	45	20	25	
Positive	9	7	2	
Perineural invasion				1
Negative	48	24	24	
Positive	6	3	3	

*CEA, carcinoembryonic antigen; TNM, Tumor-Node-Metastasis stage*.

Further, we evaluated the expression levels of miR-372 and CADM2 in GC and normal tissues, and the differential expressions were in accordance with those of TCGA (Figures 6F,G). The miR-372 expression level remarkably augmented after ADAMTS9-AS2 knockdown in MKN-45 cells and attenuated after ADAMTS9-AS2 overexpression in HGC-27 cells (Figure 6H). However, the CADM2 level was down-regulated in ADAMTS9-AS2 knockdown MKN-45 cells, and up-regulated in ADAMTS9-AS2 overexpressing HGC-27 cells as determined by qRT-PCR and western blotting (Figures 6I,J).

The miR-372 binding sites in ADAMTS9-AS2 and CADM2 3′UTR were predicted by miRcode and TargetScan (Figure 6K). Dual-Luciferase reporter assay revealed that miR-372 mimic significantly inhibited the relative luciferase activity of ADAMTS9-AS2-Wt, but the ADAMTS9-AS2-Mut was unaffected (Figure 6L). Meanwhile, luciferase reporter assay was performed for CADM2 in HGC-27 cells with ADAMTS9-AS2 overexpressing and MKN-45 cells with ADAMTS9-AS2 knockdown (Figure 6M). The results showed that the luciferase activity of wild-type CADM2 3′ UTR plasmid reduced remarkably in response to miR-372 mimic, but co-transfection of miR-372 mimic and the mutated CADM2 3′ UTR plasmid had no effect on luciferase activity. A FISH analysis was conducted in MKN-45 cells to determine the localization of ADAMTS9-AS2 and miR-372. The result showed that miR-372 co-localized with ADAMTS9-AS2 in the cytoplasm (Figure 6N). Moreover, we determine the expression levels of ADAMTS9-AS2 and CADM2 with qPCR after transfection of miR-372 mimic. As expected, the CADM2 expression significantly decreased after miR-372 overexpression. Interestingly, the expression level of ADAMTS9-AS2 was not affected (Figure 6O).

## Discussion

Thanks to high-throughput sequencing technology and the rapid development of bioinformatics, we are able to discover the various aberrant expression of RNAs in cancer cells. Unlike classic molecular or cellular biology studies that focus on a specific molecular interaction, the ceRNA network is constructed to provides a more comprehensive view of the RNA regulatory mechanism during GC carcinogenesis.

As compared to other databases that contain only the expression profile of a certain type of RNA, TCGA has the apparent advantage that it contains a series of sequencing information of lncRNA, miRNA, and mRNA of the same batch of clinical samples. This enables researchers to discover changes between different kinds of RNA and to uncover the regulatory mechanisms between the ceRNAs.

The construction of a ceRNA network depends on the expression profile characteristics of TCGA data on one hand, and the algorithm of the predictive databases on the other hand. It is unfeasible for a current database to include all RNA-RNA interaction information. Hence, some of the potential interactomes may not be included in our network, and some important regulatory information might be lost. Furthermore, some predictive databases predict RNA-RNA interactomes by simply comparing base complementary sequences of miRNA response element (MRE) and are therefore not highly reliable. In order to verify the application value of our established ceRNA network, we specially selected one of the regulatory axes for verification. Based on the results of differential expression analysis, correlation analysis, survival analysis, and RNA-RNA interaction prediction, a potential ADAMTS9-AS2/miR-372/CADM2 regulatory axis which well befitted the ceRNA pattern, was proposed.

Next, we conducted *in vitro* experiments to verify whether the actual interaction was consistent with the prediction of bioinformatics analysis. Dual luciferase reporter assay indicated that miR-372 could bind to both ADAMTS9-AS2 and 3′UTR of CADM2. The FISH assay showed that ADAMTS9-AS2 co-localized with miR-372 in the cytoplasm of GC cells. Knockdown or overexpression of ADAMTS9-AS2 could cause the corresponding expression changes of miR-372 and CADM2. These direct evidences confirm our bioinformatics predictions.

Seven of 41 lncRNAs in the ceRNA axes were significantly correlated with survival, and could serve as prognostic markers for GC. Among them, ADAMTS9-AS2 may serve as key regulators and prognostic markers in GC. The lncRNA ADAMTS9-AS2 is the antisense transcript of tumor suppressor ADAMTS9, and it is considered as a novel tumor suppressor. Previous studies have showed that expression of ADAMTS9-AS2 was downregulated in various cancers (30–34). Additionally, ADAMTS9-AS2 expression was associated with the poor survival outcomes of these cancers. Cao et al. indicated that ADAMTS9-AS2 may inhibit GC development by activating the PI3K/Akt pathway (32). A recent study suggested that upregulated ADAMTS9-AS2 inhibits lung cancer progression and promotion of TGFBR3 via suppression of miR-223-3p (33). Yao et al. found that ADAMTS9-AS2 inhibited glioma migration (34). In our research, ADAMTS9-AS2 expression was underexpressed in GC tissue. In concord with the results of previous studies, our analysis found that low expression of ADAMTS9-AS2 was implicated with poor survival outcomes. Therefore, ADAMTS9-AS2 can also serve as a significant biomarker for GC.

Several studies have been carried out on the construction of ceRNA networks for gastric cancer which majorly focused on certain lncRNAs, and were not experimentally validated (8, 11, 13, 14). Our study emphasizes the establishment of a regulatory network rather than analyzing individual genes. We selected one of the axes for in-depth research and confirmed it through experiments on clinical tissues and cell lines. Moreover, we believe that there are likely to be other important regulatory pathways in the ceRNA network we built, and detailed studies are warranted to discover these underlying mechanisms in the future.

In summary, a ceRNA regulatory network and axes were constructed to provide a comprehensive view of underlying mechanism for the progression of GC. We proposed a potential regulatory axis that ADAMTS9-AS2 might regulate the expression of CADM2 by sponging miR-372. The hypothesis was experimentally validated in both cell lines and clinical samples. We suppose that the ADAMTS9-AS2/miR-372/CADM2 axis may exert a critical role in the pathogenesis of GC.

## Data Availability

All datasets generated for this study are included in the manuscript/Supplementary Files.

## Ethics Statement

Written informed consent was obtained from all patients and the study was approved by the ethics committees of the BJH.

## Author Contributions

HP, CG, JP, DG, and SS conceived and designed the experiments. HP, CG, and JP analyzed the data and drafted the manuscript. DG and SS discussed and contributed to the data analysis. DX and YZ contributed to the sampling. All authors read and approved the final manuscript.

### Conflict of Interest Statement

The authors declare that the research was conducted in the absence of any commercial or financial relationships that could be construed as a potential conflict of interest.
