# Supplementary material for: Construction of a Competitive Endogenous RNA Network and Identification of Potential Regulatory Axis in Gastric Cancer
Source: Front Oncol. 2019 Oct 4;9:912. doi: 10.3389/fonc.2019.00912 (PMC6787165; doi:10.3389/fonc.2019.00912)
Supplement: Supplementary file 1 [file Table_1.pdf]

Table S1. Primers used for amplification of targets

Gene	Forward (5'-3')	Reverse (5'-3')
ADAMTS9-AS2	TCTGTTGCCCATTTCCTACC	CCCTTCCATCCTGTCTACTCTA
hsa-mir-372	CAACAGAAGGCTCGAGCAACCTGCG	TTCTGATCAGGATCCCATTACAGCCAGAC
	GAGAAGATAC	GCTGTAAG
CADM2	AAACTTCCAAGGCATATCTCACC	TGCGATTTGCATCCTCTTCTT
GAPDH	TGACTTCAACAGCGACACCCA	CACCCTGTTGCTGTAGCCAAA
U6	CGAGCACAGAATCGCTTCA	CTCGCTTCGGCAGCACATAT
